# Development of the Self-Perceived Safety of Orthopedic Post-Surgery Inpatients (SPSOPSI) Scale

**DOI:** 10.3390/healthcare10122343

**Published:** 2022-11-22

**Authors:** Pei-Jung Yu, Lee-Ing Tsao, Chieh-Yu Liu

**Affiliations:** 1 Department of Nursing, Mackay Medical College, New Taipei City 25245, Taiwan; 2 School of Nursing, National Taipei University of Nursing and Health Sciences, Taipei City 112303, Taiwan; 3 Department of Health Care Management, National Taipei University of Nursing and Health Sciences, Taipei City 112303, Taiwan; 4 Department of Teaching and Research, Taipei City Hospital, Taipei City 10341, Taiwan

**Keywords:** patient safety, self-perceived safety, orthopedic, validity, reliability

## Abstract

In recent decades, patient safety in orthopedics has gained increasing importance and has been regarded as a core concept of medical care quality. However, according to currently published studies, measurement instruments used to evaluate post-surgery orthopedic patient’s perceptions are still very rare. This study aimed to develop a new measurement instrument, the self-perceived safety of orthopedic post-surgery inpatients (SPSOPSI) scale, which can provide healthcare workers with a better understanding of orthopedic patients’ self-perceived safety and give more precise clinical suggestions. Item analysis and exploratory factor analysis (EFA) were used, and the results showed that the six-factor model is good-fit: root mean square residual (RMR) 0.00, root mean square error of approximation (RMSEA) 0.06, goodness-of-fit index (GFI) 0.90, comparative fit index (CFI) 0.98, incremental fit index (IFI) 0.98. The results showed the SPSOPSI scale is a valid and reliable tool for health care providers can use to evaluate orthopedic post-surgery patients’ perceived safety.

## 1. Introduction

Orthopedic patients’ self-perceived safety has gained increasing importance for clinical medicine and nursing care in recent decades. Over the years, patient safety policies in various countries have also changed. Over the past few decades, most hospitals mainly focused on preventing occurrences of adverse events. There are still disputes regarding the scope of this issue. In recent years, research proposed that safety policy making should involve patients; however, understanding patients’ self-perceived safety in current policy making is still limited. The World Health Organization and the European Commission also suggested that patients should participate in the prevention of medical errors [1,2].

One article reported that the methods of measuring patient safety have been verified by systematic literature and comprehensive analysis [3]. Most existing scales or inventories only considered surface validity, and in-depth interviews or observations are still limited. A systematic review showed that most measurement tools related to patient safety are still used by medical personnel actively to evaluate safety. There is no apparent best method to measure safety in general practice [4]. Another systematic review showed that a common method to measure hospital patient injury was to assess the understanding of safety from the policy perspective of key processes through accident notification. The most used questionnaire was the Patient Safety Culture Survey. However, those surveys were rated by healthcare institutions without involving patients. Although they can be used to evaluate the patient safety of healthcare institutions, they cannot provide comprehensive safety care [5]. If healthcare institutions only focused on safety culture, when the external and internal support fails to monitor it, the institutionalized safety culture will be destroyed [6].

The concept of patient participation in the personal medical care process has been applied to various kinds of patient care. The process should be redesigned to allow patients to participate in safety to improve its quality [7]. The patient’s perception of safety is one of the key elements of future clinical care because understanding patients can help provide more appropriate safety care [8]. Nursing quality and patient safety from both patients’ and health care professionals’ perspectives showed that medical professionals rated nursing quality higher than the patients did. Therefore, health care providers need to be able to provide care according to patients’ needs [9]. A study also showed that inpatients’ perception of safety is affected by the general treatment process, trust in the medical system, nursing care, and hospital environment. Hence, more should be done to enhance patients’ safety awareness, patient centricity and its related factors [10].

In summary, patient safety has become the core concept of medical quality. However, according to currently published studies, measurement instruments involving patient’s perceptions are still very rare, especially for orthopedic patients. Even some intervention studies’ use of mHealth after orthopedic surgery has increased the quality of care [11,12]. However, the self-perceived safety of orthopedic post-surgery inpatients has not been specifically studied before. Therefore, developing a new measurement instrument of safety involving patient’s perceptions is urgently needed. This study aimed to develop a new measurement instrument, the self-perceived safety of orthopedic post-surgery inpatients (SPSOPSI) scale, which can provide healthcare workers with a more comprehensive understanding of orthopedic patients’ perceived safety and give more precise clinical suggestions.

## 2. Materials and Methods

This study adopted a cross-sectional study design, mainly in the development of the SPSOPSI scale and to test its validity and reliability.

### 2.1. Development of Items

The scale developed in this study was based on qualitative research first, including in-depth inter-views with participants, word-by-word coding, translation, analysis, and induction, and forms a theoretical base to further develop questions of SPSOPSI. The preparation procedure of the scale mainly refers to the practice of Devellis [13] and is divided into the following eight stages: (1) To clearly define the concept of hypothetical measurement, after coding and analyzing the qualitative data of in-depth interviews, literature verification is also adopted to summarize the conceptual analysis of postoperative patients’ perceived safety; (2) To establish an item pool using the researcher’s many years of practical experience in orthopedic nursing and the data collection and analysis results of the first stage qualitative research; (3) To determine the format for measurement, consider whether the question is consistent with the research purpose, is the appropriate question type, uses simple and easy-to-understand words, clearly defines each question, covers only one idea, avoids subjective and emotional words, and has an appropriate sentence length; (4) Reviewed by experts to determine the appropriateness and clearness of the initial items through calculation of the content validity index (CVI) of each question, scored by experts; (5) Consider inclusion of validity item by including a group of socially expected questions in the scale; (6) Administer items to a development scale. The community of variables is more than 0.7. Based on Comrey’s study [14], the proportion between the number of items in the scale and the number of pre-tests is about 1:5 to 1:10. Considering the inferential statistical analysis, factor analysis and exploratory factor analysis will require a large sample, and these were 10 times the number of the scale questions; (7) Evaluation of the items. We used statistical methods to analyze the goodness-of-fit of the test questions. We took the item analysis, validity test, and reliability test of the pre-test questionnaire as the basis for preparing the formal questionnaire and deleting items in the scale; (8) Rationalization of scale length. The scale should not unduly burden the participants but have good reliability. We also considered the balance between parsimony and reliability when developing the scale.

### 2.2. Participants

The study participants were recruited from orthopedic wards of one quasi-medical center in northern Taiwan. The inclusion criteria are: (1) inpatients diagnosed with orthopedic and need to receive surgery; (2) aged elder than 20 years old; (3) able to communicate in Chinese or Taiwanese; (4) able to be interviewed by researchers. Content validity index (CVI) was calculated by using expert content validity, experts agree with the content of measurement tools by quantitative means. A newly developed scale or inventory needs to be greater than 0.80 before it is acceptable [15,16].

### 2.3. Item Analysis

To validate whether there is a good correlation between the items and each scale, this study carried out item analysis for each item of the scale based on the fact that the corrected item correlation between the single item and the total scale must be greater than 0.3. The critical ratio (CR) of each item was calculated and the sum of the scores of all participants in the pre-test scale was ranked from high to low. The total scores of this scale were divided into groups with the top 27% of the t-test scores as the high score group and the bottom 27% as the low score group. Regarding the significant test of the difference between the average scores of each item between the high and low groups, if the CR value of the item reached the significant level (α < 0.05), the question can identify the response of the different participants. This is the primary consideration to consider when deciding whether to delete the item [17].

### 2.4. Factor Analysis

The principal components factoring (PCF) of the exploratory factor analysis (EFA) and varimax rotation method were chosen, among the orthogonal axis methods, to take the factors with eigenvalues greater than 1 and factor loads greater than 0.5 [18], and to obtain the maximum explanatory variation to test the construct validity of the scale. The common factors among the items of the energy table were extracted to reduce and confirm the dimensions [19,20]. Before using the factor analysis, we first used the Kaiser-Meyer-Olkin (KMO) test and Bartlett’s spherical test to determine whether the scale is suitable for factor analysis. According to Kaiser [21], the larger the KMO value, the more common factors among variables, the more suitable it is for factor analysis. If the KMO value is less than 0.50, it is less suitable for factor analysis.

Confirmatory factor analysis (CFA) was used to analyze the statistical method of construct validity of SPSOPSI. For the confirmatory factor verification of model fit analysis, when evaluating the overall fit of the model, based on Hair et al. [22] model fit index to find out the best goodness-of-fit to the SPSOPSI. Several criteria were used to evaluate the goodness-of-fit of the model, including normed chi-square value, standardized root mean square residual (SRMS), root mean square error of approximation (RMSEA), goodness-of-fit index (GFI), comparative fit index (CFI), and incremental fit index (IFI).

### 2.5. Reliability

To evaluate the consistency of participants’ performance on the test questions, Cronbach’s alpha was used to test the internal consistency of each item under the same structure to compare the homogeneity between questions. Statistically, the internal consistency coefficient is between 0.7–0.8. If it is lower than 0.35, the test questions should be modified [23]. The intraclass correlation coefficient (ICC) is used to indicate the same group of participants answering the same scale, and then the scores of the previous and subsequent tests are correlated by product difference. To measure the correlation between the two tests, 0.7–0.9 is high correlation, 0.4–0.6 is medium correlation, and below 0.3 is low correlation [24]. The descriptive statistics of continuous variables were mean and standard deviation (SD), case number (n), and percentage (%). Item analysis and exploratory factor analysis (EFA) were conducted using IBM SPSS software version 22.0 and confirmatory factor analysis (CFA) was conducted using LISREL version 8.54.

## 3. Results

The study was from 1 April to 30 August 2017. A total of 262 eligible orthopedic inpatients were recruited. The finalized version of the SPSOPSI scale comprised 20 items, which adopted the 5-point Likert scoring method, from 5 “very safe,” to 1 “very unsafe.” This scale belongs to the score summation scales, and the items of the same dimension are scored by summation. We evaluated the attitude response (degree of agreement) of each respondent. The sum of the scores in these items was the safety attitude of the respondent toward the aspect. The finalized version of the SPSOPSI is provided in Appendix A. Table 1 shows the characteristics of basic demographic data. Most of the study patients were aged >65 years (43%), female (66%), elementary school (37.8%), married (64.4%), Buddhist (66.8%), retired (61.5%), couples living together (40.5%). A total of 29.0% of the patients underwent two or four operations and 96.6% of the patients were accompanied. Almost all patients suffered from more than one chronic disease (98.4%).

We consulted orthopedic experts and senior practitioners, including seven experts (orthopedists, patient safety experts, and senior nursing staff) to confirm whether the contents of the question items are consistent with the research purpose and concept. The content validity score was based on the Polit and Beck’s study [25], in which the I-CVI (item-level content validity index) was lower than 0.80 as the standard for deleting questions. Two items were thus deleted, and the experts suggested adjusting the content of two items. Finally, the overall scale S-CVI (scale-level content validity index) index of 24 items was 0.96. A total of 24 items analysis show that the value of correlation coefficient is significant between 0.27–0.68 (*p* < 0.05), indicating that 21 items are homogeneous with the total scale and have discriminating power. The correlation values of the other three questions ranged from 0.27 to 0.29 (*p* > 0.05) and were thus deleted. Therefore, after the CR value of item analysis and correlation analysis, the scale had 21 items, which are listed in Table 2.

The 21 items to be retained were confirmed to be of commonality by EFA. The Bartlett test was used to obtain the correlation of each variable in the sample correlation matrix, which is 3231.304 (*p* < 0.001), indicating a high correlation between these topics. The KMO value is 0.90, indicating that the research data sampling is appropriate. After the varimax rotation of principal component analysis, 11 items were classified to the first factor, 3 items in the second factor, 3 items in the third factor, and 3 items in the fourth factor, and then we deleted the fifth element with only one item, and the factor loading was 0.26. Finally, 20 questions and four factors were extracted and listed in Table 3, and the total variance explained is 90%.

**Figure 1:**
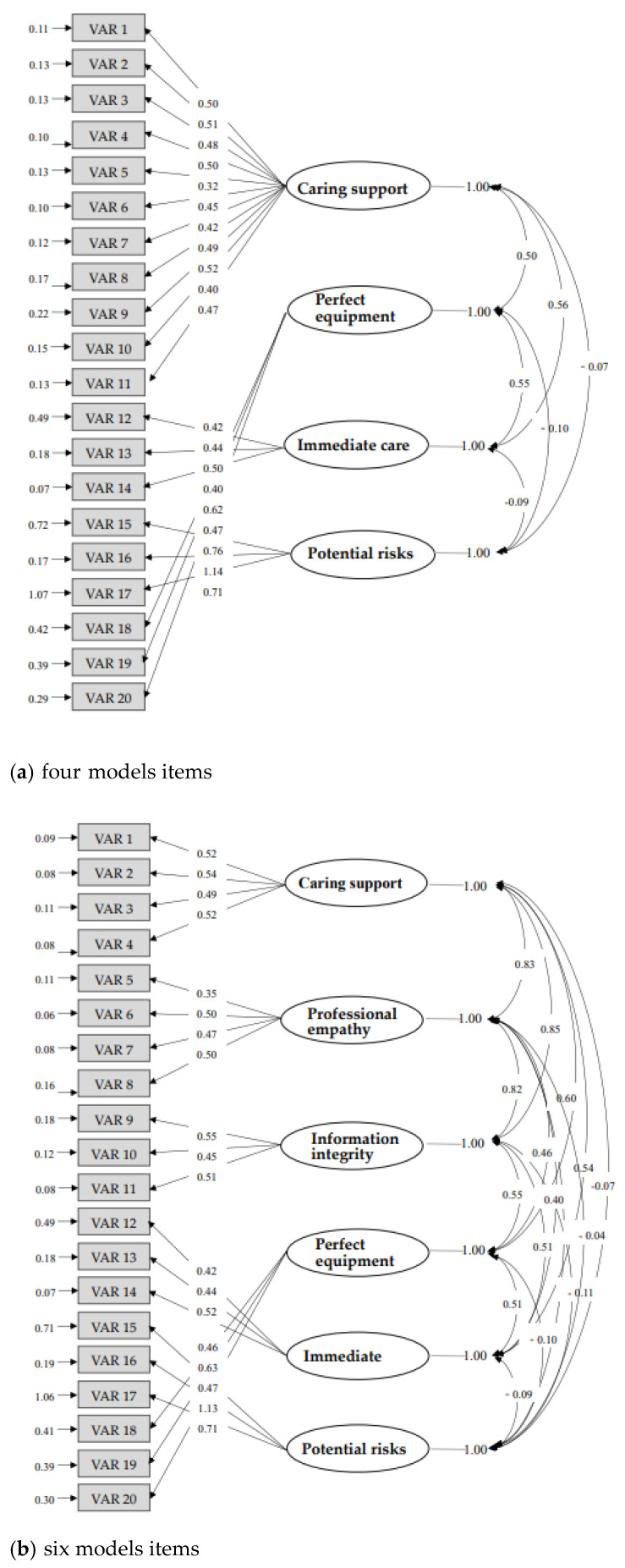
Confirmatory factor analysis for four (**a**) and six (**b**) models for items in the self-perceived safety.

According to the model specification, this study constructed a path diagram based on the assumptions in the theoretical model, and finally converted the path diagram into a series of structural and measurement equations to reconfirm the appropriateness of the scale structure. In this study, the hypothesis model of the SPSOPSI scale was obtained by item analysis and EFA, and 20 items of the four-factor model for the initial qualitative research items were created. The structural appropriateness of the two models is shown in Figure 1. After reviewing the four-factor model, the original first factor, entitled ‘caring support’ with 11 items were further rearranged into three new subscales: ‘caring support’ (item 1–item 4), ‘professional empathy’ (item 5–item 8) and ‘information integrity’ (item 9–item 11).

Comparing the goodness-of-fit indices between four-factor and six-factor models, the results showed that the six-factor model outperformed the four-factor model, which showed that normed chi-square value was 1.95 < 3.20 for the six-factor model, the root mean square residual (RMR) was almost 0.00 < 0.05 for the six-factor model, the root mean square error of approximation (RMSEA) was 0.06 < 0.09 for the six-factor model, goodness-of-fit index (GFI) was 0.90 > 0.83 for the six-factor model, CFI was 0.98 > 0.96 for the six-factor model, and incremental fit index (IFI) was 0.98 > 0.96 for the six-factor model, as listed in Table 4. Therefore, the six-factor model of SPSOPSI was confirmed.

Reliability of Cronbach’s α of 20 items of the SPSOPSI scale of the six factors to test the internal consistency of the same construct. The results show that the coefficient α of the six factors were 0.92 for “care support,” 0.89 for “professional empathy,” 0.86 for “information integrity,” 0.68 for “equipment improvement,” 0.76 for “immediate care,” and 0.78 for “potential risk,” respectively. The coefficient α is 0.85 for the entire scale, and for each sub-scale the coefficient α is still above 0.65, indicating good internal consistency (Table 5). The intraclass correlation coefficient of the scale was tested in 30 cases two weeks after the first measurement, and the calculation was verified by ICC. The results show that “care support” is 0.98, “professional empathy” is 0.99, “information integrity” is 0.97, “equipment improvement” is 0.72, “immediate care” is 0.85, and “potential risk” is 0.94. The ICC of each sub-scale is more than 0.70, which is listed in Table 5, indicating good test–retest reliability.

## 4. Discussion

This study developed a new scale of measuring the self-perceived safety of orthopedic post-surgery inpatients, the SPSOPSI scale, which originated following the creation of a theoretical base through qualitative study and further established the connections between concepts and item generation to form the question items. The results showed that the validity, reliability, and internal consistency of the SPSOPSI scale can effectively evaluate the self-perceived safety of orthopedic post-surgery patients. The combination of exploratory and confirmatory factor analysis benefits more accurately explains the usefulness of one newly developed scale [26]. This study followed the rationale of alternative models (AM), as proposed by Jöreskog and Sörbom [27], who defined several alternative theoretical models or competitive models and collected a group of empirical data to test which theoretical model fits best. Confirmatory factor analysis (CFA) is suggested to be the preferred method to check the model in a multi-dimensional scale, and the model goodness-of-fit index of the theoretical model constructed by CFA is the best way to investigate the validity and reliability of one newly developed scale [28].

Based on the qualitative research, this study established a solid concept, first, by constructing an ideal path diagram, and finally, by transforming the path diagram into a series of structural and measurement equations to form the SPSOPSI scale. This study used the four factors of the results of EFA and the six factors of the scale developed from the concept and theory of qualitative research and adopted the “competitive model” test goodness-of-fit measurement in CFA to compare the model fit indices. In the CFA, when evaluating the overall fitness of the model, according to the comparison table of the two model fitness indicators, an RMSEA of less than 0.08 was considered an acceptable fitness. The RMSEA of the six-factor model (RMSEA = 0.06) in this study was better than the four-factor model (RMSEA = 0.09), and the goodness-of-fit index (GFI) was 0.9 for the six-factor model, which was higher than the four-factor model (GFI = 0.83). Therefore, the six-factor model was regarded as a better and more suitable model, and finally, the six-dimensional SPSOPSI scale was adopted.

Previous research points out that more attention should be paid to the relationship between the path model and latent variables in the model evaluation process [29]. Our research has scientifically verified the evolution from the four dimensions of EFA to the six dimensions of CFA. The change is limited to the 11 items in “adequate support” in the first dimension of the four dimensions scale, which is divided into four items in “care support” and “professional empathy,” and three items in “information integrity” in the first three dimensions of the six dimensions scale. The original 11 items in “adequate support” were further discussed. The 11 items in the qualitative research stage belong to the interactive category. After data analysis by means of induction, comparison, and contrast, new ideas are developed, and concept maps are used to illustrate the relationship between theoretical concepts.

According to the literature, patients know little about safety and how situations affect patient safety during hospitalization [30]. In the past, no in-depth discussions have taken place to understand how patients interpret their safety and their perceived role in safety [31]. The research shows that interviewing patients’ feelings and experiences regarding patient safety are considered to determine various topics affecting clinical practice. Participants’ concept of safety focuses on realizing high standards of medical care within the trust relationship. The main factors that may affect patient safety include the patient’s attitude, behavior, and health awareness; thus, understanding the patient’s views and experiences is important in inpatient safety management [32]. Care support, professional empathy, and information integrity are the subcategories of the concept maps, which are integrated into “Adequate support,” the main category. Therefore, the statistical analysis results confirm that the items of the scale developed based on the theory have a good correlation with each scale, and truly reflect the samples. Thus, the six factors in the SPSOPSI scale were better constructed based on qualitative research and also showed better goodness-of-fit than the four-factor model, which can be considered a well-constructed scale.

Scales related to patient safety have utilized different types of tools over the past 20 years, and many experts have sorted out different forms of systematic literature and published them in the literature. Through systematic literature verification and comprehensive analysis, Aneesh et al. [3] compared more than 20 kinds of patient safety scales. The scales focus mainly on three main dimensions, including a patient safety attitude questionnaire, a patient safety culture questionnaire, and a patient safety atmosphere questionnaire. Colla et al. [33] also checked the systematic literature to understand the use of patient safety-related scales. Nine scales are commonly used to evaluate patient safety, all of which are scored with the 5-point Likert scale. The attributes of the scales are divided into two categories, one for managers and the other for staff, and the dimensions of these scales are similar. Based on the study of the existing patient safety experience and results scale in the systematic literature, Ricci Cabelloa et al. [32] determined 28 research reports. There are 23 different patient safety-related tools, 15 written questionnaires for self-response, six for telephone interviews, and two for electronic reporting system. Most tools focus on specific aspects of patient safety, and the most common emphasis was on the experience of adverse drug reactions, and the conclusion is put forward. Although there is evidence that most patient safety scales have psychometric characteristics of considerable reliability and validity, the published scales did not consider the patients’ self-perceived safety, though the SPSOPSI scale did.

## 5. Conclusions

In conclusion, the results showed that the SPSOPSI scale had very good reliability and validity to use to evaluate orthopedic post-surgery patient’s perceived safety. The SPSOPSI instrument can also help health care providers as it is an appropriate tool for assessment and evaluation to provide more precise clinical care to inpatients with different diseases.

## 6. Limitation

This study has some limitations. The first limitation is the sample characteristic limitation. In this study, we only included orthopedic patients who were almost elderly. Another limitation is the smaller sample sizes, though we used proper data sampling and statistical methods throughout this study.

## Figures and Tables

**Table 1:** Patient demographic characteristics (n = 262).

Characteristics	n	%
**Age**		
<30 yr	20	7.6
31–40 yr	7	2.7
41–50 yr	23	8.8
51–64 yr	87	33.2
>65 yr	125	47.7
**Gender**		
Male	89	34
Female	173	66
**Education level**		
Elementary school	99	37.8
Middle school	34	13.0
High school	69	26.3
College	48	18.3
Graduate school	12	4.6
**Marital status**		
Married	168	64.1
Unmarried	37	14.1
Divorced	19	7.3
Widowers and widows	38	14.5
**Religion**		
Buddhism	175	66.8
Protestant	10	3.8
Catholic	2	0.8
Daoism	40	15.3
Others	34	13.0
**Occupation**		
Farmers	29	11.1
Business	17	6.5
Public employees	12	4.6
Service	43	16.4
Retired	161	61.5
**Living status**		
Living alone	33	12.6
Living as couple	106	40.5
Living with children	89	34.0
Living with relatives	23	8.8
Other	11	4.2
**Accompanied**		
Yes	253	96.6
No	9	3.4

**Table 2:** Item analysis of the SPSOPSI scale (n = 262).

Item	Mean	SD	Correlation to Total Scale
Q1	4.42	0.60	0.67 **
Q2	4.39	0.61	0.68 **
Q3	4.48	0.60	0.68 **
Q4	4.42	0.59	0.68 **
Q5	4.71	0.48	0.54 **
Q6	4.60	0.55	0.65 **
Q7	4.55	0.54	0.62 **
Q8	4.46	0.64	0.63 **
Q9	4.34	0.70	0.60 **
Q10	4.56	0.56	0.58 **
Q11	4.44	0.59	0.66 **
Q12	3.93	1.17	0.38 **
Q13	4.40	0.82	0.35 **
Q14	4.55	0.61	0.48 **
Q15	4.53	0.58	0.49 **
Q16	2.00	1.01	0.27
Q17	3.49	1.14	0.30 **
Q18	2.97	1.21	0.35 **
Q19	2.98	1.25	0.33 **
Q20	4.04	0.79	0.41 **

** *p*-value < 0.01.

**Table 3:** Composition matrix after the varimax rotation of the SPSOPSI scale (n = 262).

Item	1	2	3	4	5
Q1	0.81				
Q2	0.77				
Q3	0.73				
Q4	0.78				
Q5	0.74				
Q6	0.86				
Q7	0.82				
Q8	0.80				
Q9	0.72				
Q10	0.71				
Q11	0.73				
Q12					0.85
Q13			0.78		
Q14			0.72		
Q15			0.76		
Q16				0.86	
Q17				0.90	
Q18				0.64	
Q19		0.73			
Q20		0.75			
Q21		0.72			

Note: Items with factor loading >0.5 are retained.

**Table 4:** Indicator of goodness-of-fit model.

Model	X^2^/df	SRMR	RMSEA	GFI	CFI	IFI
I	3.20	0.05	0.09	0.83	0.96	0.96
II	1.95	0.00	0.06	0.90	0.98	0.98

Abbreviations: **X^2^/df**, normed chi-square; root mean square residual (RMR), root mean square error of approximation (RESEA); goodness-of-fit index (GFI); comparative fit index (CFI); IFI, incremental fit index.

**Table 5:** Reliability analysis of the SPSOPSI scale (n = 262).

Factor	Number of Questions	α Coefficient	Intraclass Correlation Coefficient (ICC)
Caring support	1–4	0.92	0.98
Professional empathy	5–8	0.89	0.99
Information integrity	9–11	0.86	0.97
Perfect equipment	18–20	0.68	0.72
Immediate care	12–14	0.76	0.85
Potential risks	15–17	0.78	0.94

## Data Availability

Not applicable.

## Appendix A

**Table A1:** Self-Perceived Safety of Orthopedic Post-surgery Inpatients Scale (SPSOPSI).

Subscale and Items	NS	LS	SS	WS	FS
A. Professional empathy					
1. Health care workers can empathize with me.	□	□	□	□	□
2. Health care workers can listen to me.	□	□	□	□	□
3. Health care workers care about my feelings.	□	□	□	□	□
4. Health care workers value my needs and aid.	□	□	□	□	□
B. Professional empathy					
5. I feel that the physicians are treating me professionally.	□	□	□	□	□
6. I feel that the nursing staff takes care of me professionally.	□	□	□	□	□
7. I feel that staff are aware of patient safety.	□	□	□	□	□
8. I feel that staff can adjust or make changes to how they care for me.	□	□	□	□	□
C. Information integrity					
9. I know that health care workers communicate clearly and understand my condition.	□	□	□	□	□
10. I can understand when the physicians explain the condition to me.	□	□	□	□	□
11. I can understand the care provided by the nursing staff.	□	□	□	□	□
D. Immediate Care					
12. At any time, family, friends, or caregivers are by my side.	□	□	□	□	□
13. The attending physician visits me daily.	□	□	□	□	□
14. The nursing staff visits me regularly.	□	□	□	□	□
E. Potential risks					
15. I often worry about pain when moving.	□	□	□	□	□
16. I often worry about falling.	□	□	□	□	□
17. I often worry about mistakes that may occur.	□	□	□	□	□
F. Perfect equipment					
18. I see the toilet floor is often dry.	□	□	□	□	□
19. I see that the hospital has provided solid and stable facilities.	□	□	□	□	□
20. I feel safe during hospital stays.	□	□	□	□	□

NS: Not Safety; LS: Little Safety; SS: Some Safety; WS: Well Safety; FS: Full Safety.
